# Supplementary material for: “Es Muy Tranquilo Aquí”: Perceptions of Safety and Calm among Binationally Mobile Mexican Immigrants in a Rural Border Community
Source: Int J Environ Res Public Health. 2022 Jul 9;19(14):8399. doi: 10.3390/ijerph19148399 (PMC9323766; doi:10.3390/ijerph19148399)
Supplement: Supplementary file 1 [file ijerph-19-08399-s001.zip › File S1.pdf]

Interview Guide:
Guía de Entrevista:

Study Title: Factors of Stress and Resilience Among Mexican Origin Adults in Rural Border Communities of Southwestern Arizona
Título del Estudio: Factores de Estrés y Superación entre Adultos de Origen Mexicano en Comunidades Rurales y Fronterizas del Suroeste de Arizona

Introduction: First, thank you very much for taking the time to speak with me today. Our research team from the University of Arizona and *Campesinos sin Fronteras* really appreciates your participation in this study. In this study, we hope to learn about important mental and physical health concerns for people in your community. In addition, we are interested in learning about the major forms of stress in your community and how people cope with and overcome that stress and how they feel it may impact their health. The results of this study will be used to develop more effective health programs and guide community actions to reduce the effects of stress in Yuma County.

I appreciate your willingness to participate in this interview via telephone. I would have liked to complete this interview with you in person but would like to recognize the importance of keeping everyone safe during the COVID-19 pandemic.

I would like to ensure that you spoke with the promotora about the project and she provided you with a consent document. Also, as a reminder participation in this study is completely voluntary. You may choose to stop the interview at any time or choose to not answer any specific question as it arises. In addition, anything you share with me during this interview is completely confidential; I am also in a confidential location at this moment.

Before we begin, do you have any questions for me?

Introducción: Muchas gracias por tomar tiempo para hablar conmigo hoy. Nuestro equipo de investigación de la Universidad de Arizona y *Campesinos sin Fronteras* agradece mucho su participación en este estudio. Por medio de este estudio, esperamos aprender acerca de las preocupaciones importantes de la salud física y mental en su comunidad. Además, estamos interesados en aprender sobre las fuentes principales del estrés en su comunidad y las maneras en que la gente maneja y supera el estrés y como piensa que este puede afectar su salud. Los resultados de este estudio se utilizarán para mejorar los programas de salud y para enfocar acciones comunitarias que reduzcan los efectos del estrés en el condado de Yuma.

Agradezco su disposición a participar en esta entrevista por teléfono. Me hubiera gustado completar esta entrevista con usted en persona, pero me gustaría reconocer la importancia de mantener la salud y seguridad durante la pandemia de COVID-19.

Me gustaría asegurarme que habló con la promotora sobre el proyecto y ella le proporcionó un documento de consentimiento. Además, como recordatorio, su participación en este estudio es completamente voluntario. Puede optar por parar la entrevista en cualquier momento o optar por

no responder a cualquier pregunta específica. Además, todo lo que comparta conmigo durante esta entrevista es completamente confidencial; actualmente también estoy en un lugar confidencial.

¿Antes de empezar, tiene usted alguna pregunta para mí?

I. Background Information:
Información Personal:

What is your first name?:
¿Cuál es su primer nombre?:

What is your gender?:
¿Cuál es su género o sexo?:

In what year were you born?:
¿En qué año nació usted?:

Are you: single/married/domestic partnership/divorced/separated/widowed
Es usted: soltero/casado/con pareja/divorciado/separado/viudo

Where were you born: (city or town and state)?:
¿Donde nació usted: (ciudad o pueblo y estado)?

Do you have children: If so, how many? What are their ages?
Tiene usted hijos: ¿Si es que si, ¿cuántos? ¿Qué edades tienen?

How many are living with you now?
¿Cuántos viven con usted ahora?

Do you have family members who live on the other side of the border from where you live?
¿Tiene usted familiares que viven al otro lado de la frontera de donde usted vive?

Who else lives in the home with you?
¿Quién más vive en la casa con usted?

Do you work outside the home: If so, what is your employment?
Trabaja usted fuera de la casa: ¿Si es que si, a que se dedica usted?

How long have you had that job, and has it been affected by the COVID 19 pandemic?
¿Por cuanto tiempo ha tenido ese trabajo? Ha sido afectado por la pandemia de COVID-19?

Does anyone else in your home work, and if so what is their employment?
¿Hay alguien más en su casa que trabaja y si es que si, a que se dedica esa persona?

II. Concepts of Health, Stress and Resilience
Conceptos de la Salud, el Estrés, y la Superación

To start, what does being healthy mean to you?
Para empezar, ¿Que significa, para usted, el estar sano o tener salud?

What are the most important things someone can do to be healthy?
¿Cuales son las cosas más importantes que puede hacer uno para estar sano?

What do you think makes a person unhealthy?
Qué piensa usted que causa la mala salúd?

Do you think that people need to see doctors in order to stay healthy?
¿Usted piensa que la gente necesita acudir con los médicos para mantenerse sano?

Do you consider yourself to be a healthy person? Why or why not?
¿Usted se considera a si mismo ser una persona saludable? Porque si o no?

Do you think that people in your community are generally healthy? Why or why not?
¿Usted considera que los miembros de su comunidad son generalmente saludables? Porque si o no?

III. Describing Your Community
Describiendo a su Comunidad

Where do you live now? How long have you lived there?
¿Dónde vive usted ahora? ¿Cuánto tiempo ha vivido allí?:

Why did you decide to move to that place?
¿Porqué tomó la decisión de mudar a ese lugar?

How would you describe the place you live? How is it similar or different from other places where you have lived?
¿Cómo describe usted el lugar donde vive? ¿Como se compara con otros lugares donde ha vivido?

Who are the people who you see most regularly on a day-to-day basis? Has that changed dramatically due to the COVID-19 pandemic?
¿Cuáles son las personas que usted vea con más frecuencia día a día? ¿Eso ha cambiado dramáticamente dado a la pandemia de COVID-19?

Do you regularly cross the border? If yes, is it for work, to see family, go to the doctor, go grocery shopping, or for other reasons?
Usted cruza regularmente la frontera? Para trabajar, visitar familia, ir al doctor, o ir de compras, o por otras razones?

Has crossing the border become harder for you in recent years, due to COVID-19 restrictions, or for other reasons?
¿Se ha dificultado la cruzada de la frontera para usted en los últimos años dado a la pandemia de COVID-19 o por otras razones?

What do you like about the community where you live?
¿Qué es lo que le gusta de la comunidad donde vive?

Are there things that you wish you could change about your community?
¿Hay cosas que le gustaria cambiar de su comunidad?

What are the things that make you feel safe or unsafe in your community?
¿Cuáles son las cosas que lo hacen sentir seguro o inseguro en su comunidad?

IV. Sources of Stress
Fuentes de Estrés

When you hear the word stress, what does it make you think of?
¿Cuándo escucha la palabra estrés, que significa para usted?:

We understand that the COVID-19 pandemic has been a stressful time for so many of us. How has it been for you and your family?
Entendemos que la pandemia de COVID-19 ha sido un tiempo muy estresante para tanta gente. ¿Como ha sido para usted y su familia?

Have there been significant changes in your family because of the pandemic? For example, job loss, people infected by the virus, or the inability to gather with loved ones?
¿Ha habido cambios muy grandes en su familia dado a la pandemia? Por ejemplo, la falta de empleo, personas infectadas con el virus, imposibilidad de juntarse con sus seres queridos, etc.

What are some of the things, in your life, that you miss from before the pandemic?
¿Cuáles son algunas de las cosas en su vida de antes de la pandemia que extraña?

Are you interested in getting the COVID-19 vaccine, if so, do you have access to it?
¿Está interesado en recibir la vacuna COVID-19 si es así, tiene acceso a ella?

Do you think that getting vaccinated would reduce your stress related to the pandemic?
¿Cree que vacunándose reduciría su estrés relacionado con la pandemia?

What are other sources of stress in your life aside from the pandemic?
¿Cuáles son los otros factores de estrés en su vida que no son relacionados a la pandemia?

What kinds of things cause stress for other members of your family?
¿Qué cree usted que le causa estrés á otros miembros de su familia?:

Do you think the causes of stress in a border community like Yuma County are different than in other places?
¿Cree usted que las causas del estrés en una comunidad fronteriza como el condado de Yuma son diferentes a las de otros lugares?

Do you feel that the presence of border patrol and police is a cause of stress for you or members of your family or community?
¿Usted piensa que la presencia de la patrulla fronteriza o la policia es una fuente de estrés para usted o miembros de su comunidad?

Do you believe that you have control to change or get rid of the things that cause stress in your life?
¿Piensa usted que tiene control para cambiar o eliminar las fuentes de estrés en su vida?

How do you know when you are stressed?
¿Cómo sabe usted cuando está estresado?

Do you notice or feel changes in your body when you are stressed?
¿Usted puede notar o sentir cambios en su cuerpo cuando esta estresado?

Do you think that stress is harmful to your health: How?
Usted piensa que el estrés daña la salud: ¿Cómo?

V. Coping with Stress
Superando el Estrés

What do you do to cope with or manage stress in your life?
¿Cómo se sobrepone o lidea con el estrés en su vida?

Do you think that the COVID-19 pandemic has changed the way that you cope with stress? How?
¿Usted piensa que la pandemia de COVID ha cambiado la manera en que lidea/maneja el estrés? ¿Cómo?

Would you say that these strategies are effective in lowering your stress levels?
¿Diría usted que esas acciones para bajar el estrés le sirven para bajar sus niveles de estrés?

How do you relax? Or what relaxes you?
Que le relaja? O como se relaja usted?

Can you tell me about a stressful situation you went through recently and how you got through it?
Me podría contar de una situación estresante que vivió últimamente y cómo la supero y salió adelante?

Have you learned anything during the pandemic that has helped you to overcome your hard moments?
¿Hay algo que usted ha aprendido durante la pandemia que le ha ayudado a superar sus momentos dificiles?

Is there someone or something in your life that most helps you deal with hard times?
¿Hay alguien o algo en su vida que le brinde más ayuda en sus momentos difíciles?

Is there anything that you do to reduce your stress level that you think could be bad for your health?
¿Cree usted que algo de lo que usted esta haciendo para bajar su nivel de estress podria hacer dañ su salud?

VI. Sources of Well-Being and Strength
Fuentes de Bienestar y Fortaleza

What makes you happy or brings you joy in your life right now: Why?
Qué es lo que le hace feliz o le da alegría en su vida en este momento: ¿Por qué?

What is the best part of your day: Why?
Cuál es la mejor parte de su día: ¿Por qué?

What is your favorite past-time: Why?
Cuál es su pasatiempo preferido: ¿Por qué?

What or who motivates you to overcome challenges in your life?
¿Qué o quién le motiva a salir adelante en su vida?

Is there something that you wish you could do to improve how you feel but cannot do for any reason?
Hay algo que usted quisiera hacer para sentirse mejor, pero por alguna razón no puede?

If you could change one thing in your life, what would it be?
¿Si usted pudiera hacer algún cambio en su vida, que sería?

The next phase of our study involves scientifically measuring stress in the body in order to better understand the burden of stress in this border community. Cortisol is a stress hormone that is found in people's hair and nails.

If you were asked to participate in this next phase of our study, would you be willing to provide a small sample of your hair or nails: If yes, which one would you prefer?

La siguiente fase del estudio involucrará el medir científicamente el estrés en el cuerpo para entender mejor los niveles del estrés en esta comunidad fronteriza. Cortisol es una hormona del estrés que se encuentra en el cabello y las uñas de las personas.

Si a usted se le pidiera su participación en esa próxima fase del estudio, estaría dispuesto a proveer una pequeña muestra de pelo o uñas: Si es que si, ¿cuál prefería?

Do you think other people would be willing to participate in this next phase of the study: Why or why not?
Usted piensa que otras personas estarían dispuestas a participar en esta próxima fase del estudio: ¿Porque sí o porque no?

How would you recommend that we talk about this study to members of your community?
¿Cómo recomendaría usted que hablemos sobre este estudio con los miembros de su comunidad?

Do you think this is a worthwhile study? Why?
¿Cree usted que este estudio vale la pena? ¿Porqué?

That is the end of this interview. I would like to thank you very much for speaking with me today. The information you shared with me will be very helpful for our study.

There will be additional steps to this study and there are many opportunities to participate. A CHW will follow up with you with more opportunities to be involved.

Do you have anything else you would like to share or any questions before we conclude?

Once again, thank you very much for your participation.

Hemos concluido esta entrevista. Muchas gracias por hablar conmigo hoy. La información que usted compartió nos ayudará mucho en este estudio.

Habrá pasos adicionales para este estudio y hay muchas oportunidades para participar. Una promotora se comunicará con usted para brindarle más oportunidades para participar.

¿Tiene usted algo más que le gustaría compartir o alguna pregunta para mi antes de concluir?

De nuevo, muchas gracias por su participación.
