# Supplementary material for: “Es Muy Tranquilo Aquí”: Perceptions of Safety and Calm among Binationally Mobile Mexican Immigrants in a Rural Border Community
Source: Int J Environ Res Public Health. 2022 Jul 9;19(14):8399. doi: 10.3390/ijerph19148399 (PMC9323766; doi:10.3390/ijerph19148399)
Supplement: Supplementary file 1 [file ijerph-19-08399-s001.zip › Table S1.pdf]

Table S1. Code Tree.

Stress and Resilience Interview Code Book				
**Theme**	**Code**	**Sub Code**	**Definition**	**Example**
Stress				
	Stress Reduction Techniques		Ways that the respondents describe responding to stress.	Go out into nature, Walking with the family, Sports, Looking for resources on the Internet
		Mind Body Techniques	References to practices that connect the mind and body to promote stress reduction	Meditation exercises, spending some time alone
		Negative Coping	References to activities to reduce stress that are unhealthy	Eating fast food, drinking or doing drugs
		Exercise	References to physical activity as a way to reduce feelings of stress	Going to the gym relaxes me.
	Definition of stress		Responses to what stress means to the respondents	something out of control in your life, poor emotional health, mental fatigue
	Manifestations of stress		Ways that stress is experienced in the body and mind	Feeling pressure, headache, feeling of anxiety
	Economic stress		Concerns about finances	being able to work and support the family

	Family Problems		Stress or problems produced within the family context	Divorce
	Immigration/border stress		Stressors related to immigration status, legal issues, long lines, enforcement.	family divided between the US and Mexico
	Stress -Health Link			
	Covid stress		The ways that COVID exacerbated stressors	Missed medical appointments, closed churches, job loss, illness
		Covid Vaccine	Mention or reference to the COVID-19 Vaccination	receiving doses, vaccine hesitancy
	Other stress			
Resilience				
	Faith/Spirituality		References to the role of religion and spirituality in one's life	essential for well-being, calm and relaxation
	Positive attitude		Optimism and positive orientation toward life and the inevitable problems that one is confronted with	be thankful for what we have
	Family support		References to how the family is a source of happiness, motivation, strength	The family is a source of happiness; deep dependence on family support

	Perseverance/ goal orientation		References to plans for the future that give one hope in spite of challenges	get up day after day clinging to dreams of finishing school, getting ahead
	Social support		Providing and receiving help from people other than family members	Spending time with or talking to a loved one (friend or family)
	Sources Joy/Past-Times		When people talk about best part of their day, what makes them happy, feel alive, etc.	Finishing days work and coming home to family. Going out to do activities that are fun.
	Inspiration for Future		Motivation for the future and what drives it	Children and parents are inspiration, aspiring to complete goals
	COVID resilience		Positive responses to the stress caused by COVID.	during COVID immediate family members became more interdependent, closer together
Health				
	Community Health		assessment of the health of individuals who live in the community	Noticing people drinking, neighbors sharing about medical conditions like diabetes
	Definition of Health		How health is defined by participants.	Absence of illness, feeling strong physically, having energy, ability to work
	Healthy behaviors		References to how to live a healthy life	food/nutrition, physical activity, sleep, good relationships, strength
	Own Health		When speaking about their assessment of their personal health	describing health conditions: high cholesterol, poor diet choices, mental illness, depression

	Barriers to Health		References to difficulty in engaging in opportunities to promote healthier outcome for self	negative environmental exposures (pesticides), having to cross the border for healthcare, little access to healthy food options when eating out
	Doctor's Role		Describing medical/clinical intervention in health outcomes	Going to doctor for physical exam, going to doctor for illness, doctor providing information about overall health for own awareness
	Unhealthy behaviors		References to activities considered unhealthy and causes of ill health/health practices	poor health related to drug, alcohol or fast food use, eating red meat contributes to poor health outcomes.
Border life				
	Crossing		Anything related to border crossing not captured by subcodes	ways in which people cross (by foot or car), length of lines to cross the border, interaction with border officials, changes in border enforcement, border closures
		Reasons for Crossing	References to activities across the border	family visits, doctor, carry money
		Crossing Frequency	References to how frequently one crosses into Mexico	several times a week
	Positive Attributes		References to the positive qualities of the communities	everything is very quiet/calm
		Economic Opportunities	Progression from Mexico to San Luis AZ. to a better house	for a better life

		Calm Environment	References to border community feeling safe and stable as well as calm, tranquilo, small	walkability, friendly neighbors, feeling safe
		Community Resources	things (people, places, organizations, programs) in the community which provide support to people	low-income support, teachers, financial support for medical expenses or bills
		Mexican Enclave	References to feelings of familiarity with others in the community due to common heritage	It's strange – here there are no people like Americans, there are almost none here. There are more Mexicans.
	Police presence		Positive and negative references to the presence of police and border patrol	crossing the border, traffic control, patrolling, keeping community safe
	Binational connection		Networks with people on the other side, binational identify	what connection do we have with the line
	Desired improvements		References to how the community could be more conducive to good health	lack of infrastructure for bicycles, walks,
	COVID Impacts		Changes in border life since COVID, including both positive and negative	Time it takes to cross.

	Conditions in Mexico		Use for when people describe conditions in Mexico, especially as they compare to living in the US, in terms of economics, safety/security issues, etc.	green environment, police corruption, drugs, crime
	Mexican culture		References to their culture and how they see it	Mexican food, family ties, community engagement, work life
	Negative Attributes		Codes for anything that feels hard or negative about the border region	small town, low food options, lack of activities for kids
		Drugs/Alcohol	References to drug use, problems with drugs, drug trafficking	people using drugs selling drugs, hearing about drug violence in MX
		Dangers Border	References to safety concerns in border region	cartel activity, mass shootings, gang members
	Social Networks		Social connections with people, groups, organizations	friends, co-workers, family members, neighbors
