# Supplementary material for: “Es Muy Tranquilo Aquí”: Perceptions of Safety and Calm among Binationally Mobile Mexican Immigrants in a Rural Border Community
Source: Int J Environ Res Public Health. 2022 Jul 9;19(14):8399. doi: 10.3390/ijerph19148399 (PMC9323766; doi:10.3390/ijerph19148399)
Supplement: Supplementary file 1 [file ijerph-19-08399-s001.zip › Table S2.pdf]

Article Section	Original Quotes in Spanish
3.1	Yo pienso que lo que es – primero que nada, lo más que me gusta es que es una ciudad tranquila ¿no? No hay – no es una ciudad muy grande, no es escandalosa. (Luis)<br><br>Es más tranquilo que no se – he ido pues a California y partes así, Phoenix y se me hace como mucho tránsito. Y aquí puedo andar en la calle sin miedo de – pues de manejar porque yo soy muy nerviosa para manejar. (Manuela)<br><br>En el aspecto de tranquilidad, aquí donde yo estoy viviendo, vivo alrededor de muchas personas mayores, pues está una comunidad muy tranquila. A otros lugares, pues viví allá en Yuma y era como un parqueadero de trailers donde vivía y había mucho desorden, muchos problemas. Y aquí no, todo está muy tranquilo. (Anita)<br><br>Pues es muy pacifico, muy calmado, pues todos los vecinos míos son no, ni bronqueros, jamás he tenido bronca con nadie ahí, muy pacifico, muy calmado. (Francisca)

3.1.1	Mi comunidad aquí en Somerton es algo muy pacífico, tranquilo; no es tan estresante, porque es prácticamente un pueblo chico. Yo diría que no es tan estresante este lugar. (Adán)<br><br>No, no creo que sean iguales porque siento que tanto la comunidad como la ciudad es un poco más tranquila y hay otros lugares donde todo se mantiene más acelerado. Entonces, no creo que sea igual. (Mónica)<br><br>Y aquí yo siento que – si – disfrutas un poco más – pero – no se como explicarlo 100% pero – siento que es más tranquilo en estas áreas que allá en una ciudad. (Luis)<br><br>Mi comunidad aquí en Somerton es algo muy pacífico, tranquilo; no es tan estresante, porque es prácticamente un pueblo chico. Yo diría que no es tan estresante este lugar... en ocasiones hemos tocado el tema como de comprar ya una casa y como que, ¿para dónde? ¿A dónde nos vamos? Pues yo siempre he dicho: “no, pues Yuma no, Yuma no”, porque podría estar en algunas zonas muy bonito, en algunas zonas, muy tranquilas; pero sí se siente poquito más el estrés de vivir en la ciudad por la cuestión de tanto tráfico, quizás mucha gente maliciosa, etcétera. Y aquí sí es algo un poquito más tranquilo. Sí, es prácticamente, es como mi espacio, mi zona de confort y pues a lo mismo que – yo vivo en departamentos, o sea, no tengo casa todavía, ¿no? Y, de hecho, vivo donde trabajo. Y pues me siento cómodo, seguro aquí, ¿por qué? Porque yo sé cómo está todo a mi alrededor, pues conozco a toda la gente de aquí, los vecinos; sé cómo están las instalaciones. Entonces, no tengo mucho de qué preocuparme. (Olger)<br><br>Si, yo creo que sí. Yo creo que llevar una vida con bastante estrés si te va como deteriorando, como acabando. (Manuela)
3.2.1	Andan los de inmigración aquí atrás de la casa, anda el helicóptero desde temprano ... y pues no puedes saber si vaya a haber –que alguien vaya corriendo o traiga un arma. Pues nunca ha pasado porque pues no creo que vengan a hacer estas cosas, ellos cruzan porque quieren progresar. Pero igual, ha habido muchos casos de que ellos no vienen armados, pero los mismos policías pueden hacer un tiroteo nomás por querer agarrar a alguien. (Rosie)<br><br>“The violence, like cartels doing like killings and gun shootings across the border ... like that type of like news has been increasing and that like the only worry is like, when will it spill over to this side?” (Chucho, English speaker)

3.2.2	"Ahorita hay mucha delincuencia y eso dicen que es mucho basado en que todos quieren tomar las fronteras. Ahorita hay mucho – se ha mirado mucho en San Luis rio Colorado – hay mucha – pues algo que nunca se había mirado acá, asesinatos, ya secuestros a la luz del día." (Ana)<br><br>Oh, que – pues sí, es muy diferente a la comparación actualmente a donde vivía en México porque allá si era, pues, donde yo vivía si era una cuadra muy peligrosa, había muchas personas drogándose o nomás simplemente salir al – porque yo vivía en una esquina y nomás salir a mi casa podía que alguien te asaltara, porque muchas veces llegó a pasar así, no a nosotros, pero sí a otra gente. (Diego)
3.2.3	Pues mira, la comunidad donde estamos es tranquilo, hasta eso. Pero si se refiere a México, México es más movido. En Mexicali todo es movimiento, movimiento, movimiento. Todo lo que es Yuma County es muy tranquilo. Aquí hay más personas mayores, entonces es más tranquilo, dentro de lo que cabe. (Mónica)<br><br>Aquí en San Luis, Arizona me siento muy segura. No hay, como se puede decir como en México, tantas pandillas, tantos adicciones, tantos alcohol—tanta gente en la calle mal habida pues. No – me siento muy segura, muy segura aquí, más pacifica, puedo – si puedo dejar un ratito a mis niñas afuera mientras entro a tomar agua o así algo, tengo mi cerco y todo, pero las puedo dejar un ratito afuera," (Elia)<br><br>Casi no se escucha que hay problemas. Ahorita hay muchos accidentes de carro, pero osea no se escucha de que hubieron balazos, cosas así feas, no se escuchan. (Angeles)<br><br>Aquí puede uno ir a gusto a llevar a los niños al parque o a sacarlos a caminar. Así es, bien raro cuando se oye algo mal aquí en San Luis, Arizona, que se oiga algo de que mataron a alguien aquí o atropellaron o algo, allá no, allá no, allá te le quedas mirando mal a alguien y no la haces. (Francisca)

3.2.4	Todas las familias se dedican solamente a los de ellos. (Tomás)<br><br>Yo, yo la verdad siento una comunidad segura, aquí donde estoy me siento segura, tan segura que esto está mal de mi parte, que a veces digo yo dejé – dejó la bicicleta afuera y sin el foco prendido de afuera “Ahí amanece la bicicleta.” [Risa]. … La bicicleta, sí. Entonces yo me siento como que “ay,” como que “si me la van a cuidar los vecinos,” (Sabina)<br><br>Lo que lo hace diferente es que está llegando gente nueva, joven, de México. Entonces, un poco más la cultura de México y se asientan aquí y se puede decir que por eso hacemos como un match, porque tenemos prácticamente las mismas raíces y el mismo comportamiento. (Tomás)<br><br>También cuidamos de que – como si ven algo raro, pues por ahí avisa o también si una mascota se salió y la encontraron en tal parte, pues, también avisan y así. Estamos como que muy bien comunicados. (Diego)

3.2.5	Pues realmente no, creo que es una, un medio de seguridad para la comunidad y para los que están a nuestro alrededor en los dos lados de la frontera. (Francisca)<br><br>Pues me hace sentirme segura el control de la policía, que si, siento que si hacen su trabajo bien, según yo verdad. Y este pues que le puedo decir, pues no se, estoy muy agusto aquí. (Malena)<br><br>Si, si es tranquilo, me siento segura. Y luego tengo vecinos policías aquí.Tengo vecinos policías le digo, tengo cinco vecinos policías. (Ivis)<br><br>Pues me hace sentir más seguro porque aquí tenemos a 30 segundos el departamento de policía y bomberos. Eso me hace sentir muy seguro porque – pues no porque vivamos aquí no van a dejar de pasar cosas ¿verdad? pero eso nos da mucha tranquilidad. (Adan)<br><br>Seguro, a mí me hace sentir seguro, porque sí hay muy buena vigilancia, siempre, siempre. A pesar de que vivo por la main, por la pura principal, todos los días a la misma hora pasa una patrulla. Que, o sea, me toca estar en la casa, pasa una patrulla todos los días a la misma hora, siempre dando vueltas. (Pancho)<br><br>Creo que yo, yo me siento protegida en esta comunidad, creo que el departamento de policía hace bien su trabajo, pienso que cuando tienen necesidad de llamarlos ahí están asistiéndolos, porque no es una comunidad muy grande. (Sabina)<br><br>Siento que me hace sentir seguro de que que aquí en San Luis, Arizona puedes marcar al 911 por una emergencia y responden muy rápido. Los oficiales aquí son si son muy equipados, son muy entrenados profesionales. (Ernesto)<br><br>“Pues últimamente lo que se ha visto es que han mandado más, ¿cómo se puede decir? Más soldados, guardia nacional, cosas así que se ha dicho que hay más movimiento de lo que es la seguridad pública ¿no? O de las fuerzas armadas y de hecho pues ya te sientes un poquito más a gusto porque, o sea, ya como que cuando empezaron a llegar va bajando lo que es el índice de inseguridad ¿no?” (Luis)

3.3	La tranquilidad. Me siento, le voy a poner un ejemplo. Me siento libre de tener los vidrios de mi carro abajo sin estar volteando a mi alrededor. Me siento tranquila yo aquí. Agusto. (Ami)<br><br>En cuestiones de tranquilidad, antes de la pandemia podía irse uno a los parques y no es ningún problema, no hay vandalismo descontrolado. (Tomás)<br><br>Lo bueno es que tiene – que uno puede caminar muy a gusto por la calle, los carros no andan rápido, te respetan las señales, te respetan todo, está muy bien. (Mario)<br><br>Pero le comento de que – ah pues sí, es simplemente eso de que yo me he sentido muy cómodo cuando salgo. Regreso y a veces digo ay la puerta quedó abierta. Ay que hubo mira, que paz se siente irte y saber que incluso no puedes ni ponerle candado, pues porque sabes que todo va a estar bien. (Evaristo)<br><br>Veo que ya es como, no, sí me siento segura y tan segura de que a veces yo me tengo que ir a un entrenamiento y eso, y a veces se me olvida prender el foco y yo no regreso con miedo, no regreso con "oh, está oscura mi casa, déjame hablarle a alguien a ver si me acompaña para venir a casa." No, yo con toda la seguridad del mundo abro la puerta de mi casa, prendo el foco, (Sabina)

3.3.1	Pues que es – aquí la comunidad es muy pacífica, donde nosotros vivimos casi no se escucha como mucho, así como en otras partes que ya robaron o que se ve mucha pandilla, así casi no. Es como – a pesar de que aquí no es buena la situación en cuanto al trabajo y el pago, pero sientes que dentro de lo que cabe es bueno para criar a tus hijos. (Belem)<br><br>Un poquito más de vida, más esa parte, tal vez mexicana o tal vez de otro tipo de cultura, pero casi, casi vienes aquí a Somerton y haz de cuenta que, como que todo mundo está encerrado, todo mundo está enclaustrado. Incluso, antes del COVID, toda la gente estaba encerrada en sus casas. (Linda)<br><br>Si estaba un millón de veces mejor como el lugar donde vivía antes, pero la verdad, sí era como un poco más como el ambiente ahí alrededor, entonces yo creo que ese es el factor más importante que me hace, que aunque yo sé que está como mejor allá, aun así siga como prefiriendo estar aquí. (Vanessa)<br><br>Es un pueblo tranquilo. Si quiere vivir tranquilidad – pienso que es un pueblo tranquilo . Lo comparo con San Luis Río Colorado, pues yo puedo decir, por tranquilidad y seguridad yo me quedo en Arizona. (Sabina)
